# Mild hypoxia triggers transient blood–brain barrier disruption: a fundamental protective role for microglia

**DOI:** 10.1186/s40478-020-01051-z

**Published:** 2020-10-28

**Authors:** Sebok K. Halder, Richard Milner

**Affiliations:** San Diego Biomedical Research Institute, 10865 Road to the Cure, Suite 100, San Diego, CA 92121 USA

**Keywords:** Brain, Blood vessels, Microglia, Chronic mild hypoxia, Blood–brain barrier integrity, Fibrinogen, Angiogenesis, Endothelial proliferation

## Abstract

We recently demonstrated that when mice are exposed to chronic mild hypoxia (CMH, 8% O_2_), blood vessels in the spinal cord show transient vascular leak that is associated with clustering and activation of microglia around disrupted vessels. Importantly, microglial depletion profoundly increased hypoxia-induced vascular leak, implying that microglia play a critical role maintaining vascular integrity in the hypoxic spinal cord. The goal of the current study was to examine if microglia play a similar vasculo-protective function in the brain. Employing extravascular fibrinogen leak as an index of blood–brain barrier (BBB) disruption, we found that CMH provoked transient vascular leak in cerebral blood vessels that was associated with activation and aggregation of Mac-1-positive microglia around leaky vessels. Interestingly, CMH-induced vascular leak showed regional selectivity, being much more prevalent in the brainstem and olfactory bulb than the cerebral cortex and cerebellum. Pharmacological depletion of microglia with the colony stimulating factor-1 receptor inhibitor PLX5622, had no effect under normoxic conditions, but markedly increased hypoxia-induced cerebrovascular leak in all regions examined. As in the spinal cord, this was associated with endothelial induction of MECA-32, a marker of leaky CNS endothelium, and greater loss of endothelial tight junction proteins. Brain regions displaying the highest levels of hypoxic-induced vascular leak also showed the greatest levels of angiogenic remodeling, suggesting that transient BBB disruption may be an unwanted side-effect of hypoxic-induced angiogenic remodeling. As hypoxia is common to a multitude of human diseases including obstructive sleep apnea, lung disease, and age-related pulmonary, cardiac and cerebrovascular dysfunction, our findings have important translational implications. First, they point to a potential pathogenic role of chronic hypoxia in triggering BBB disruption and subsequent neurological dysfunction, and second, they demonstrate an important protective role for microglia in maintaining vascular integrity in the hypoxic brain.

## Introduction

Compared to other organs, blood vessels in the central nervous system (CNS) have uniquely high electrical resistance and low permeability, which is thought to protect sensitive neural cells from potentially harmful components in the blood [1, 18, 33]. In the brain, this is referred to as the blood–brain barrier (BBB), and loss of BBB integrity is a common feature of a broad spectrum of neurological conditions, including meningitis, ischemic stroke, multiple sclerosis (MS), and CNS tumours [2, 10, 15, 22, 34]. Increasing evidence suggests that BBB integrity also deteriorates with age [5, 14], which may in part, predispose to age-related vascular dementia. With this in mind, there is currently an intense research effort aimed at gaining a greater understanding of the molecular basis of the BBB, why it breaks down under disease conditions, and what cellular and molecular mechanisms are in place to promote repair of disrupted cerebral blood vessels.

Interestingly, blood vessels of the CNS are acutely responsive to changes in metabolic supply and demand placed by neural tissue. For instance, rodents exposed to chronic mild hypoxia (CMH, typically 8–10% O_2_) mount a strong vascular remodelling response throughout the brain and spinal cord, leading to 50% enhancement of vessel density over a period of 2 weeks [23, 24]. Over the same time course, cerebrovascular expression of tight junction proteins is strongly increased, suggestive of enhanced vascular integrity [16, 27]. The downside of vascular remodelling is that during the angiogenic process, endothelial cells temporarily uncouple from their neighbours as they proliferate and migrate to extend the capillary network, running the potential risk of triggering transient vascular leak. In a recent study, we demonstrated that CMH promotes transient vascular leak in blood vessels of the spinal cord [17]. We also made the key observation that vascular leak in the spinal cord is associated with aggregation and activation of Mac-1-positive microglia around disrupted vessels, and importantly, that microglial depletion profoundly increases hypoxia-induced vascular leak in the spinal cord, demonstrating an important protective role for microglia in maintaining vascular integrity in the hypoxic spinal cord [17]. In light of the importance of an intact BBB in the maintenance of cerebral health, and the occurrence of hypoxia in a wide range of different diseases (sleep apnea, lung disease, and age-related cardiac and cerebrovascular dysfunction), the goal of the current study was to answer the following questions. First, is BBB integrity compromised during exposure to mild hypoxia; second, if vascular integrity is disrupted, does this phenomenon display regional vulnerability; and third, do microglia also play a vasculo-protective function in the brain?

## Materials and methods

### Animals

The studies described were reviewed and approved by the Explora Biolabs Institutional Animal Care and Use Committee at San Diego Biomedical Research Institute (SDBRI). Wild-type female C57BL6/J mice obtained from Jackson Laboratories were maintained under pathogen-free conditions in the closed breeding colony of SDBRI.

### Chronic hypoxia model

Female wild-type C57BL6/J mice, 8–10 weeks of age, were housed 4 to a cage, and placed into a hypoxic chamber (Biospherix, Redfield, NY) maintained at 8% O_2_ for periods up to 14 days. Littermate control mice were kept in the same room under similar conditions except that they were kept at ambient sea-level oxygen levels (normoxia, approximately 21% O_2_ at sea-level) for the duration of the experiment. Every few days, the chamber was briefly opened for cage cleaning and food and water replacement as needed. During these times, mice would be briefly (< 5 min) exposed to normoxic conditions before resumption of hypoxic conditions.

### Elimination of microglia

PLX5622 was provided by Plexxikon (Berkeley, CA) under Material Transfer Agreement and formulated in AIN-76A standard chow by Research Diets (New Brunswick, NJ) at a dose of 1200 p.p.m. (1200 mg PLX5622 in 1 kg chow). In microglial depletion experiments, mice were fed a PLX5622 diet for 7 days prior to them being placed in the hypoxic chamber to deplete microglia before mice were exposed to hypoxic conditions. Once in the hypoxic chamber, these mice were then maintained on the PLX5622 diet for 7 days. Consistent with the findings of others, in these studies we found no obvious behavioral alterations, weight loss or signs of illness in mice fed a PLX5622 diet for 3 weeks [8, 12].

### Immunohistochemistry and antibodies

Immunohistochemistry was performed on 10 µm frozen sections of cold phosphate buffer saline (PBS) perfused tissues as described previously [31]. Rat monoclonal antibodies from BD Pharmingen (La Jolla, CA) reactive for the following antigens were used in this study: CD31 (clone MEC13.3; 1:500), MECA-32 (1:100), and Mac-1 (clone M1/70; 1:100). The hamster anti-CD31 (clone 2H8; 1:500) and rabbit anti-Tmem119 (clone 28-3; 1:200) monoclonal antibodies were obtained from Abcam (Cambridge, MA). The anti-Ki67 mouse antibody (1:500) was obtained from Vector Laboratories (Burlingame, CA). Rabbit antibodies reactive for the following proteins were also used: occludin and ZO-1 (1:2000 from Invitrogen, Carlsbad, CA), fibrinogen (1:2000 from Millipore, Temecula, CA) and aquaporin-4 (AQP4) (1:10,000 from Alomone Labs, Jerusalem, Israel). Secondary antibodies used (all at 1:500) included Cy3-conjugated anti-rabbit, anti-rat and anti-mouse and Cy5-conjugated anti-rabbit from Jackson Immunoresearch, (West Grove, PA) and Alexa Fluor 488-conjugated anti-rat, anti-hamster, and anti-rabbit from Invitrogen (Carlsbad, CA).

### Image analysis

Images were taken using a 2 ×, 10 × or 20 × objective on a Keyence 710 fluorescent microscope. For each antigen in all analyses, images of at least three randomly selected areas were taken at 10 × or 20 × magnification per tissue section and three sections per brain analyzed to calculate the mean for each animal (n = 4 mice per group). For each antigen in each experiment, exposure time was set to convey the maximum amount of information without saturating the image and was maintained constant for each antigen across the different experimental groups. The number of leaking blood vessels per field of view (FOV) was quantified by capturing images and performing manual counts of the number of vessels showing extravascular leaked fibrinogen as well as the area of vascular leakage. This method was chosen because fibrinogen staining provides a very strong and crisp fluorescent signal. The number of microglia per FOV and the percentage or number of blood vessels per FOV expressing AQP4 or MECA-32 were quantified in a similar manner. Endothelial proliferation was quantified by counting the number of CD31/Ki67 dual-positive cells per FOV. Expression of the tight junction proteins ZO-1 and occludin was quantified by measuring the total fluorescent signal per FOV and analyzed using NIH Image J software. In the analysis of microglial activity by morphological criteria, the number of microglia displaying a ramified (small cell body and long processes) or activated (large cell body and short process extensions) phenotype in specific regions were quantified and expressed as a percentage. All manual measurements were obtained in blinded manner by a single observer. Each experiment was performed with 4 different animals per condition, and the results expressed as the mean ± SEM. Statistical significance was assessed using one-way analysis of variance (ANOVA) followed by Tukey’s multiple comparison post-hoc test, in which *p* < 0.05 was defined as statistically significant. Microglial density was analyzed using Student’s t test, in which *p* < 0.05 was defined as statistically significant.

## Results

### Chronic mild hypoxia induces transient vascular leak in cerebral blood vessels that is associated with microglial accumulation and activation

**Fig. 1:**
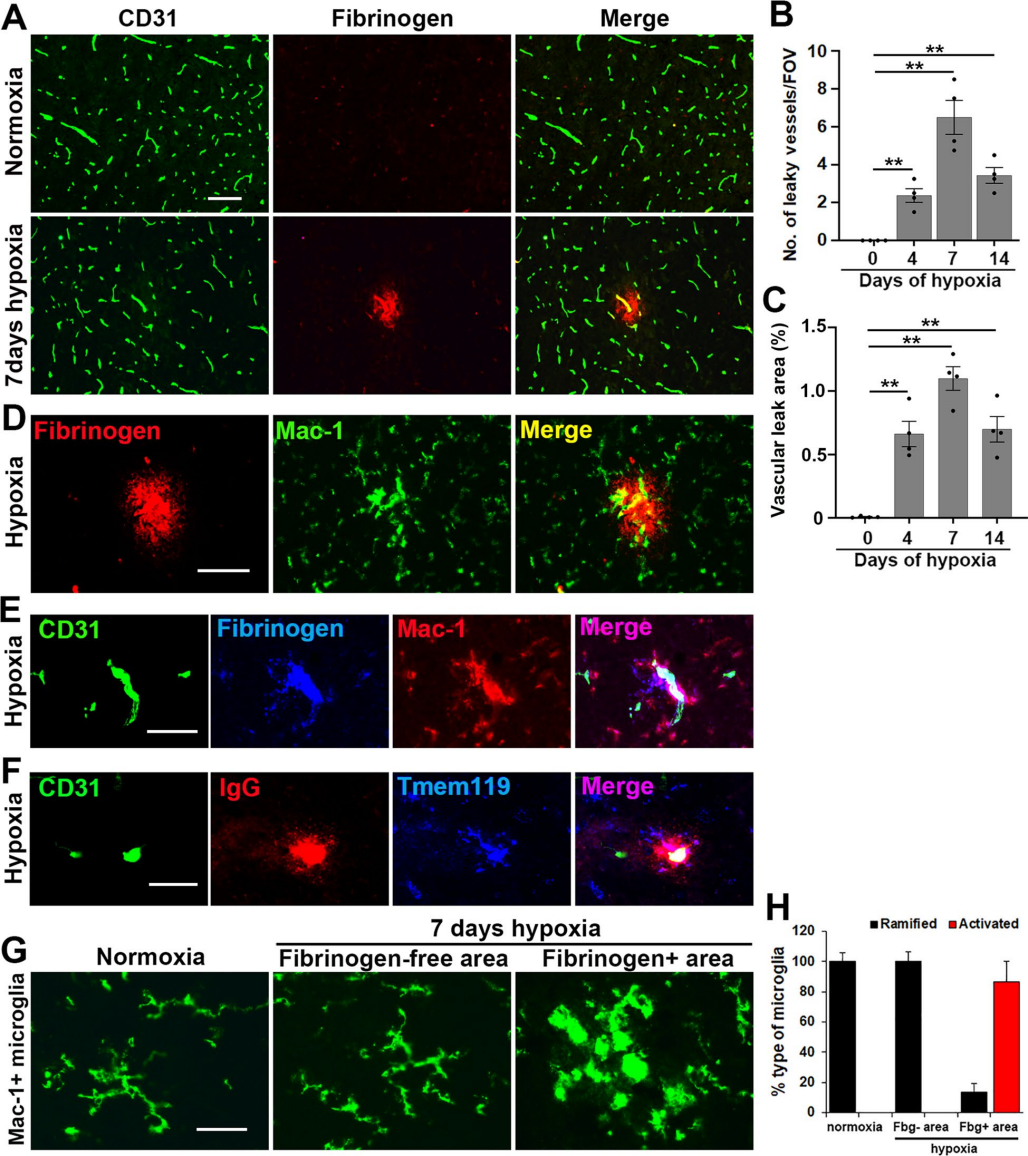
Chronic mild hypoxia (CMH) triggers vascular leak in cerebral blood vessels associated with microglial clustering. Frozen brain sections taken from mice exposed to normoxia or 7 days hypoxia (8% O_2_) were stained for the following markers: **a** the endothelial marker CD31 (AlexaFluor-488) and fibrinogen (Cy-3); **d** fibrinogen (Cy-3) and Mac-1 (AlexaFluor-488); **e** CD31 (AlexaFluor-488), fibrinogen [Cy-5 (blue)] and Mac-1 (Cy-3); **f** CD31 (AlexaFluor-488), IgG (Cy-3) and Tmem119 [Cy-5 (blue)]; and **g**, Mac-1 (AlexaFluor-488). All images were captured in the brainstem. Scale bars = 100 μm (**a**) and 50 μm (**d**–**g**). **b**, **c** Quantification of the number of leaky (fibrinogen-positive) blood vessels/FOV (**b**) or total area of vascular leak/FOV (**c**) in the brainstem after 4, 7, or 14 days hypoxia. **h** Quantification of the morphological categorization of microglia under different conditions. All results are expressed as the mean ± SEM (n = 4 mice/group). **p < 0.01 versus normoxic conditions. Note that CMH provoked transient vascular leak in brainstem blood vessels that was associated with wrapping of Mac-1/Tmem119-positive microglial processes around the damaged vessel and with a morphological switch from ramified to activated morphology (**g**)

To determine if chronic mild hypoxia (CMH, 8% O_2_) impacts the vascular integrity of cerebral blood vessels, we performed dual-immunofluorescence (dual-IF) on frozen brain sections using the endothelial cell marker CD31 and fibrinogen (Fbg) to detect extravascular leak. In the first instance, we focused our analysis in the brainstem region because it is a large well-defined area in which we’ve previously characterized hypoxia-induced vascular remodelling events [26–28]. This showed that while no vascular leak occurred under normoxic conditions, CMH induced extravascular leak in a small number of cerebral blood vessels (Fig. 1A). In a similar manner to our previous findings in the spinal cord [17], the number of leaky blood vessels/field of view (FOV) reached a peak after 7 days of CMH (*p* < 0.01 vs normoxic conditions) and declined thereafter (Fig. 1B) as did the total area of vascular leak (Fig. 1C). Dual-IF with fibrinogen and the microglial marker Mac-1 (CD11b/CD18) integrin demonstrated that extravascular fibrinogen leak was closely associated with aggregation of microglial cells around disrupted blood vessels and these microglia showed an activated morphology (larger cell body) and displayed stronger expression of the activation marker Mac-1 (Fig. 1D, E). Furthermore, CD31/IgG/Tmem119 triple-IF, using IgG as a marker of vascular leak and Tmem119 as a specific marker of microglia [3], demonstrated that blood vessel-associated Mac-1 + cells are microglia, not blood-derived monocytes (Fig. 1F). In support of this, Mac-1/Tmem119 dual-IF demonstrated strong co-localization between Mac-1 and Tmem119 (Additional file 1: Figure S1), and quantification analysis revealed that all Mac-1 + cells were also Tmem119 +. Morphological analysis revealed that under normoxic conditions or in fibrinogen-negative areas (lacking disrupted vessels) under hypoxic conditions, microglia displayed a ramified morphology (Fig. 1G). However, the majority of microglia surrounding leaky vessels under hypoxic conditions showed the typical activated morphology, with large cell body and short process extensions (shown in Fig. 1G and quantified in Fig. 1H).

### The extent of CMH-induced cerebrovascular leak is region-specific

**Fig. 2:**
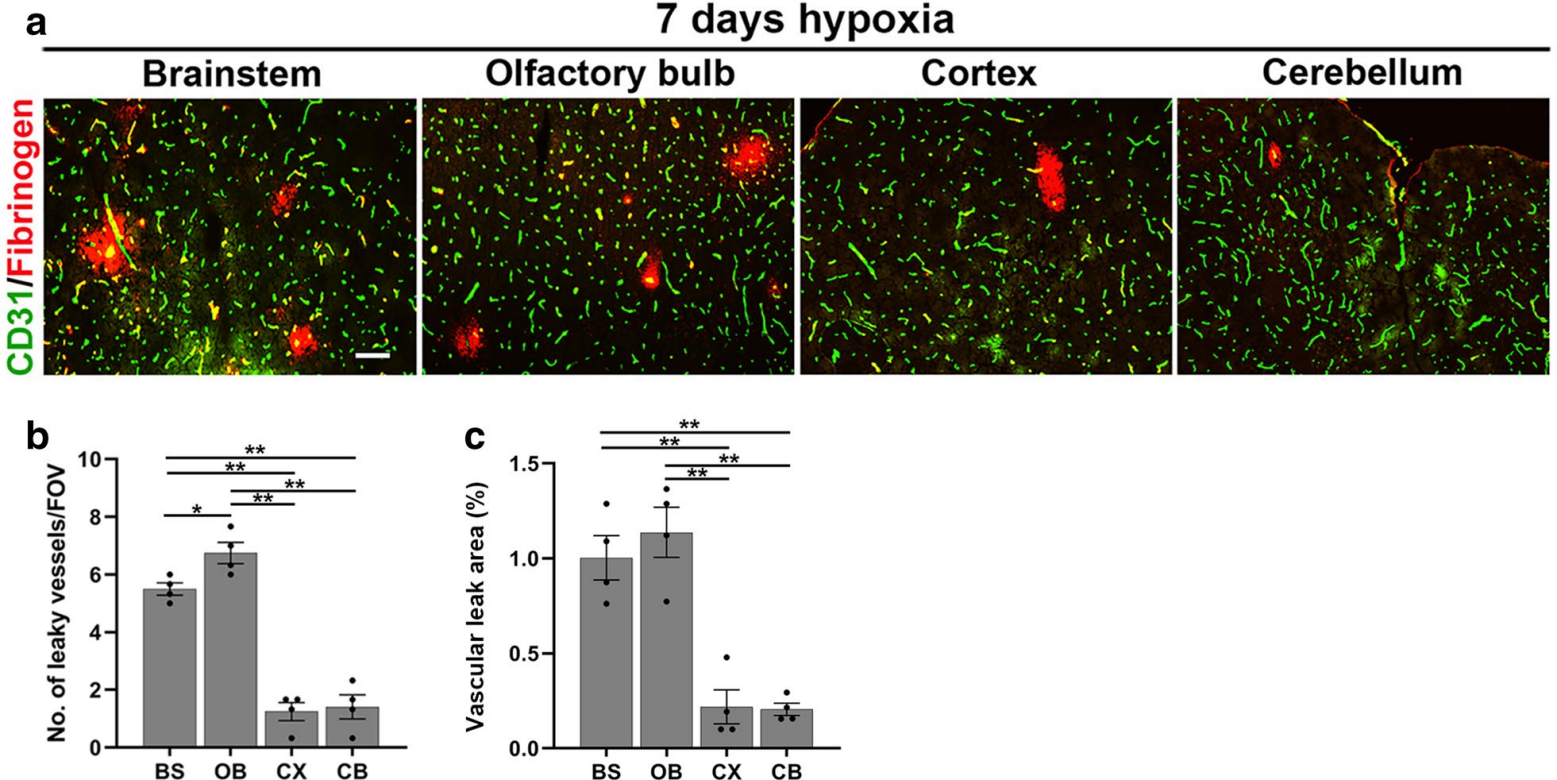
The extent of CMH-induced cerebrovascular leak is region-specific. **a** Frozen brain sections taken from mice exposed to 7 days hypoxia (8% O_2_) were dual-labelled for CD31 (AlexaFluor-488) and fibrinogen (Cy-3). Images were captured in four different brain regions: brainstem (BS), olfactory bulb (OB), cerebral cortex (CX) and cerebellum (CB). Scale bar = 200 μm. **b**, **c** Quantification of the number of leaky (fibrinogen-positive) blood vessels/FOV (**b**) or total area of vascular leak/FOV (**c**) after 7 days hypoxia. Results are expressed as the mean ± SEM (n = 4 mice/group). *p < 0.05; **p < 0.01. Note that hypoxia-induced vascular leak was significantly higher in the brainstem and olfactory bulb versus the cerebral cortex and cerebellum

Interestingly, when we examined vascular leak in different regions of the brain, we found that some regions, such as the brainstem and olfactory bulb, consistently showed the highest number of vascular leaks, while other regions, such as the cerebral cortex and cerebellum, contained far fewer vascular leaks (illustrated in Fig. 2A). Comparison of these different regions showed that the brainstem and olfactory bulb contained significantly greater numbers of leaky blood vessels as well as greater areas of vascular leak, than the cerebral cortex and cerebellum (Fig. 2B,C; *p* < 0.01).

### Microglial depletion results in greater cerebrovascular leak during hypoxic stress

**Fig. 3:**
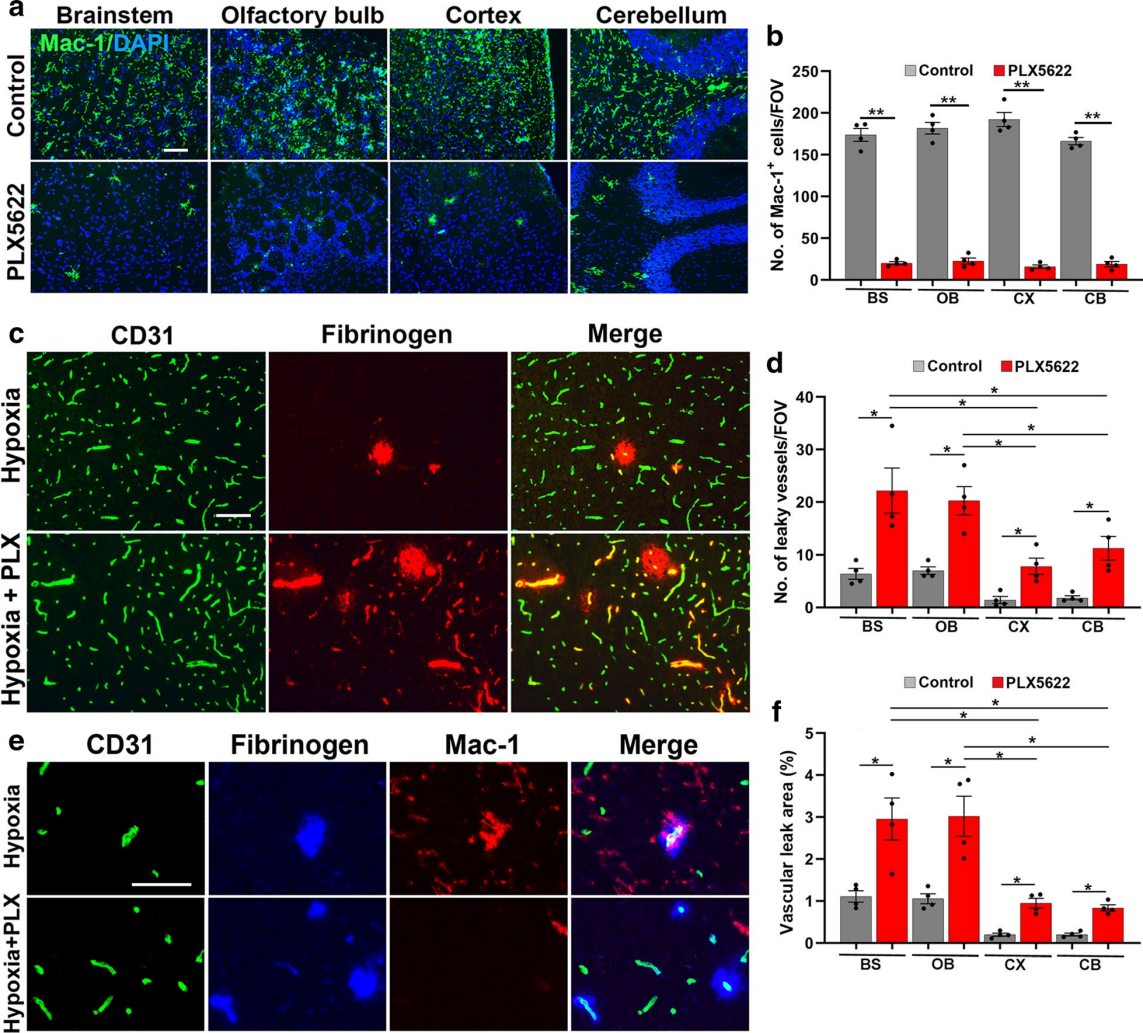
Microglial depletion results in greater vascular leak during CMH. **a** Frozen brain sections taken from mice fed normal chow or PLX5622-containing chow and maintained under normoxic conditions for 7 days were stained for the microglial marker Mac-1 (AlexaFluor-488) and DAPI. **b** Quantification of microglial depletion after 7 days PLX5622 in the brainstem (BS), olfactory bulb (OB), cerebral cortex (CX) and cerebellum (CB). Results are expressed as the mean ± SEM (n = 4 mice/group). **p < 0.01. Note that 7 days PLX5622 reduced microglial density in all brain regions examined to less than 10% of untreated controls. **c** Images of brainstem taken from mice fed normal chow or PLX5622-containing chow and maintained under hypoxic conditions for 7 days stained for CD31 (AlexaFluor-488) and fibrinogen (Cy-3). **d**, **f** Quantification of the number of leaky vessels/FOV (**d**) or total area of vascular leak/FOV (**f**) in the brainstem (BS), olfactory bulb (OB), cerebral cortex (CX) and cerebellum (CB) in mice fed normal chow or PLX5622-containing chow and maintained under hypoxic conditions for 7 days. Results are expressed as the mean ± SEM (n = 4 mice/group). *p < 0.05. Note that all regions of brain examined in PLX5622-treated mice showed a much higher number of leaky blood vessels. **e** CD31/fibrinogen/Mac-1 triple-IF of control chow mice confirmed microglial clustering and elevated levels of Mac-1 expression by microglia surrounding the leaky vessel, but absence of microglial clustering in PLX5622-fed mice. Scale bars = 100 μm (**a**, **c**) and 50 μm (**e**)

To examine if microglia in the brain behave the same as those in the spinal cord, in playing a vasculo-protective role during hypoxic exposure, we fed mice with chow containing PLX5622, an inhibitor of colony stimulating factor 1 receptor (CSF-1R), a well-established pharmacological approach of depleting microglia [8]. Mac-1 IF showed that mice treated with 1200 ppm (1200 mg drug per kg chow) PLX5622 for 7 days demonstrated greater than 90% reduction in the number of microglia seen in untreated (control) brains in all regions examined, including the brainstem, olfactory bulb, cerebral cortex and cerebellum (*p* < 0.01; Fig. 3A, B). Of note, PLX5622 treatment had no obvious effect on animal health in our studies, in keeping with the findings of others [8, 12]. To investigate the impact of microglial depletion on hypoxia-induced cerebrovascular disruption, mice were pre-treated with PLX5622 for 7 days and then exposed to CMH for 7 days, with PLX5622 maintained throughout. This revealed that PLX5622-fed mice maintained under normoxic conditions contained no leaky cerebral blood vessels (not shown). However, following 7 days hypoxia, PLX5622-fed mice showed greatly increased numbers of leaky blood vessels per FOV compared to mice fed control chow, both in the brainstem (Fig. 3C) and in the olfactory bulb, cerebral cortex and cerebellum (Additional file 2: Figure S2). Quantification confirmed that the absence of microglia resulted in a significantly increased number of leaky vessels/FOV and total area of vascular leak (both increased approximately threefold) in all four brain regions (*p* < 0.05; Fig. 3D, F). Interestingly, in microglia-depleted brains, the number of vascular leaks was still significantly greater in the brainstem and olfactory bulb compared to the cerebral cortex and cerebellum (*p* < 0.05). Triple-IF for CD31/fibrinogen/Mac-1 demonstrated microglial accumulation and increased Mac-1 expression levels on microglia close to leaky cerebral blood vessels, but lack of such responses in the brains of PLX5622-treated mice (Fig. 3E). Triple-IF with Mac-1/fibrinogen and the nuclear stain DAPI revealed that the number of microglia congregating at the leakage site ranged from 1 to 9, with an average of 4 microglia per vascular leak (Additional file 3: Figure S3).

### Under hypoxic conditions, lack of microglia results in increased expression of the leaky cerebral endothelium marker MECA-32 and region-specific astrocyte-vascular uncoupling

**Fig. 4:**
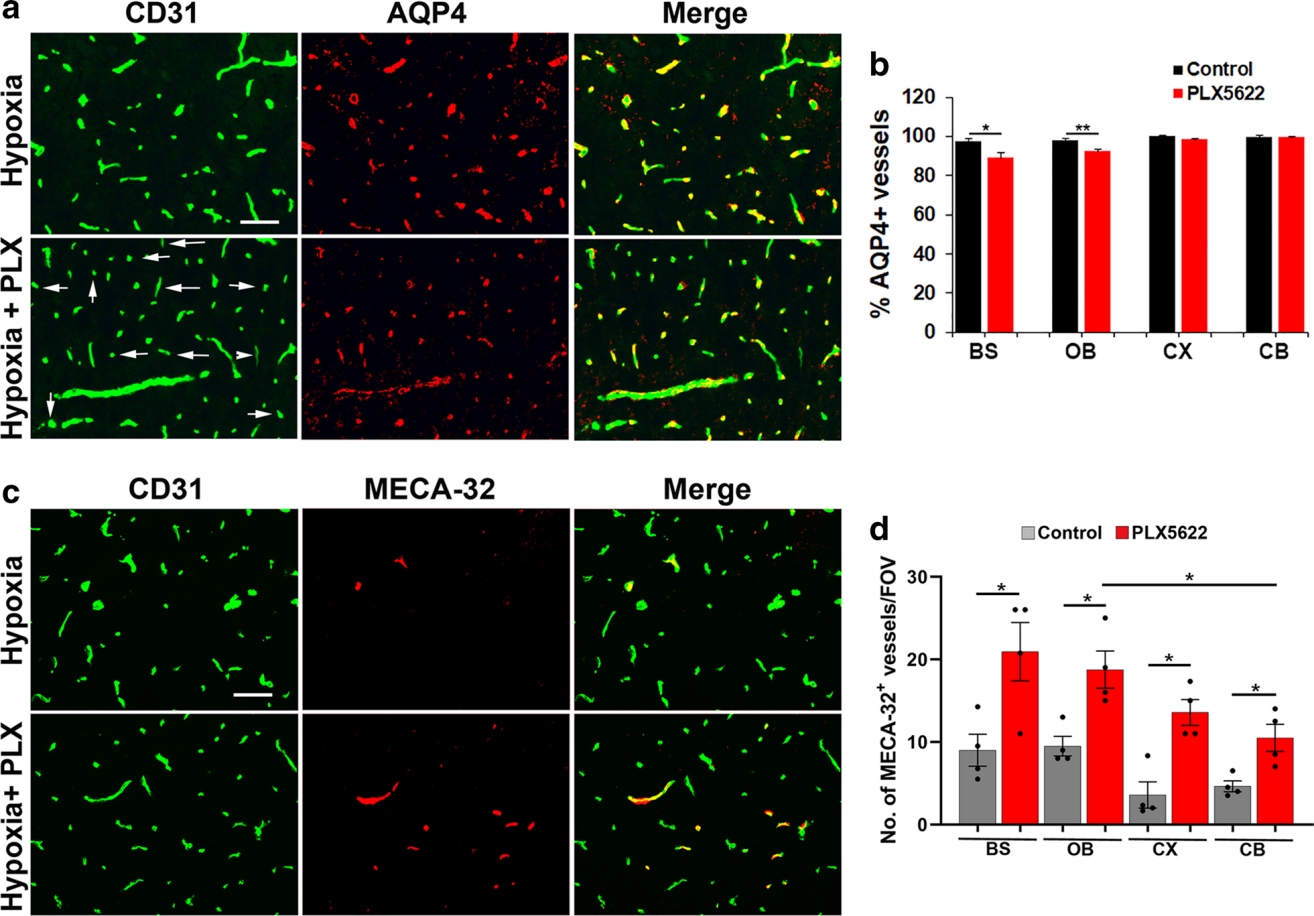
Under hypoxic conditions, absence of microglia results in increased MECA-32 expression and region-specific astrocyte-vascular uncoupling. **a**, **c**. Frozen brain sections (images captured in brainstem) taken from mice fed normal chow or PLX5622-containing chow and maintained under hypoxic conditions for 7 days were stained for CD31 (AlexaFluor-488) and AQP4 (Cy-3) in panel A or CD31 (AlexaFluor-488) and MECA-32 (Cy-3) in panel C. Scale bar = 50 μm. **b**, **d**. Quantification of the % of blood vessels expressing AQP4 (**b**) or number of blood vessels expressing MECA-32/FOV (**d**) in the brainstem (BS), olfactory bulb (OB), cerebral cortex (CX) and cerebellum (CB). Results are expressed as the mean ± SEM (n = 4 mice/group). *p < 0.05; **p < 0.01. Note that under hypoxic conditions, PLX5622-fed mice contained a significant number of cerebral blood vessels in the brainstem and olfactory bulb that lacked AQP4 expression (see arrows in **a**), though this AQP4 loss was not observed in the cerebral cortex or cerebellum (**b**). In addition, in mice fed PLX5622, all brain regions examined (brainstem, olfactory bulb, cerebral cortex and cerebellum) showed a higher number of vessels expressing MECA-32 compared with normal chow-fed controls

Astrocyte endfeet express the water channel protein aquaporin-4 (AQP4) that plays an important role in regulating vascular integrity in the CNS and provides an index of astrocyte-vascular coupling [32]. Because microglial depletion leads to greater hypoxia-induced cerebrovascular leak, we next examined whether lack of microglia affects astrocyte-vascular coupling by performing CD31/AQP4 dual-IF on brain sections taken from mice under normoxic or hypoxic conditions that had been fed control chow or chow containing PLX5622 (Fig. 4A). This revealed that under normoxic conditions, in all four brain regions examined, all blood vessels stained positive for AQP4 and lack of microglia had no obvious impact on the number of AQP4-positive blood vessels. Interestingly however, in mice treated with 7 days hypoxia, while the vast majority of blood vessels in mice fed normal chow were AQP4-positive, a significant number of vessels in the brainstem and olfactory bulb in PLX5622-fed mice showed lack of AQP4 expression (see arrows in Fig. 4A (brainstem) and in Additional file 4: Figure S4 (olfactory bulb)) and quantified in Fig. 4B (*p* < 0.05). In notable contrast, blood vessels in the cerebral cortex and cerebellum showed no obvious loss of AQP4 expression in PLX5622-fed mice (illustrated in Additional file 5: Figure S4 and quantified in Fig. 4B).

In a parallel approach to examine the impact of microglial ablation on cerebrovascular integrity in CMH-treated mice, we quantified vascular expression of MECA-32, which labels cerebral blood vessels that have reduced vascular integrity [13, 37]. This revealed that while no MECA-32 expression was detected under normoxic conditions, either in control or PLX5622-fed mice (not shown), in mice exposed to 7 days hypoxia, all four regions of the brain examined in PLX5622-fed mice showed a significantly increased number of MECA-32-positive vessels compared to those receiving normal chow (*p* < 0.05) (Fig. 4C,D and Additional file 5: Figure S5). These combined lines of evidence support the concept that when mice are exposed to CMH, absence of microglia leads to reduced BBB integrity, as shown by increased extravascular leak of fibrinogen and increased endothelial expression of MECA-32. Interestingly, in brain regions showing high vascular leak (brainstem and olfactory bulb), this correlates with astrocyte-vascular uncoupling, as defined by loss of AQP4 labelling of blood vessels, but in brain regions showing less vascular leak (cerebral cortex and cerebellum), this uncoupling is not apparent.

### In mice exposed to CMH, microglial depletion results in cerebrovascular loss of endothelial tight junction proteins

**Fig. 5:**
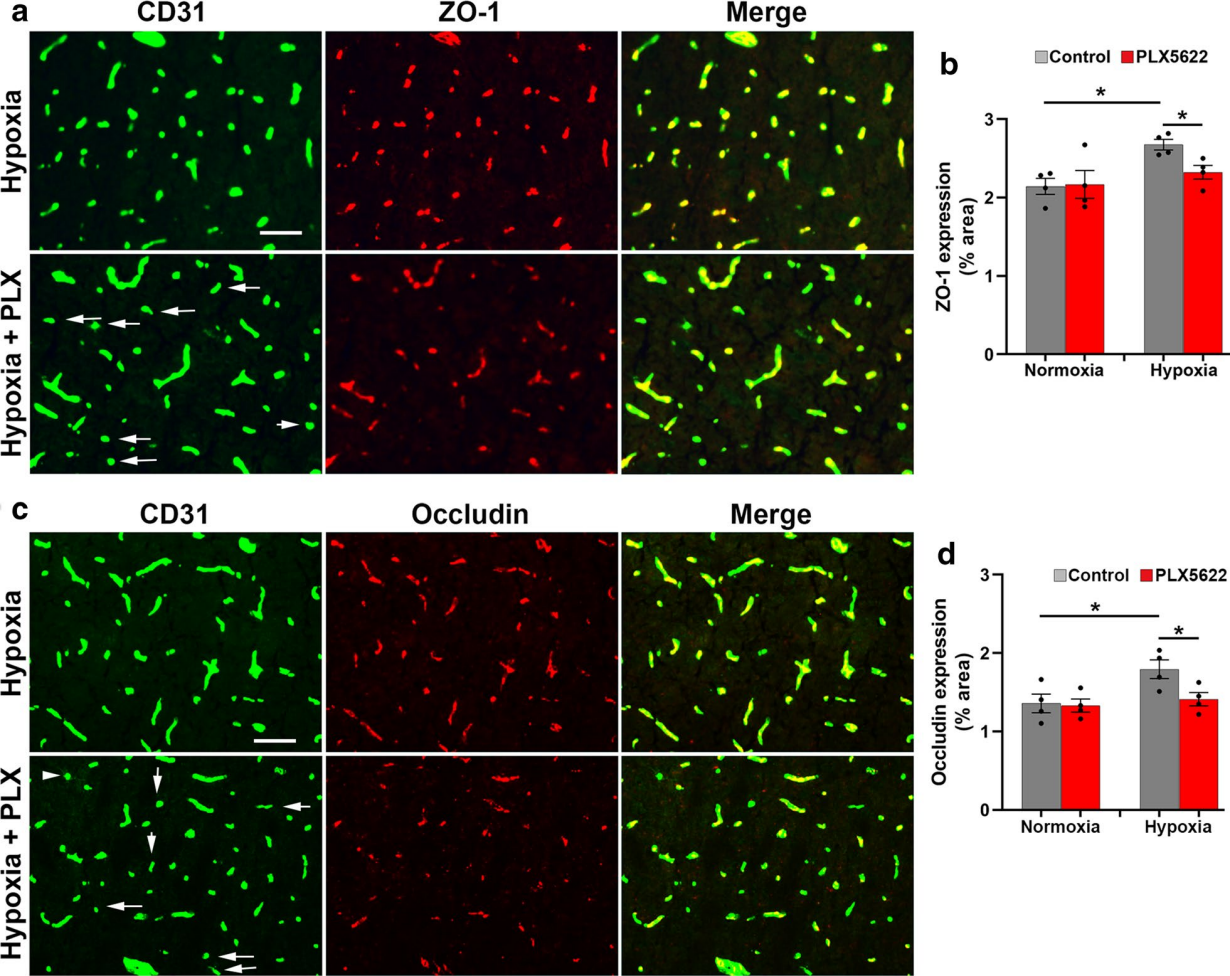
Microglial depletion results in greater loss of endothelial tight junction protein expression during CMH. **a**, **c** Frozen brain sections (images captured in brainstem) taken from mice fed normal chow or PLX5622-containing chow and maintained under hypoxic conditions for 7 days were stained for CD31 (AlexaFluor-488) and ZO-1 (Cy-3) in **a** or CD31 (AlexaFluor-488) and occludin (Cy-3) in **c**. Scale bar = 50 μm. **b**, **d**. Quantification of endothelial expression of ZO-1 (**b**) or occludin (**d**) on blood vessels in the brainstem. Results are expressed as the mean ± SEM (n = 4 mice/group). *p < 0.05. Note that under hypoxic conditions, brains of PLX5622-fed showed focal areas in which blood vessels showed diminished expression of ZO-1 and occludin (see arrows in **a**, **c**)

Endothelial tight junction proteins are a critical part of the molecular machinery responsible for the high vascular integrity observed in the CNS [1, 18, 33]; thus we next investigated if microglial absence influences cerebrovascular expression of two important tight junction proteins: ZO-1 and occludin in the brainstem region. This revealed that in mice fed normal chow, 7 days hypoxia enhanced cerebrovascular expression of the tight junction proteins ZO-1 and occludin (*p* < 0.05; Fig. 5B, D respectively), confirming previous findings [16, 27]. In contrast, PLX5622-fed mice failed to show this response, such that after 7 days hypoxia, their cerebral blood vessels expressed markedly lower levels of ZO-1 and occludin compared to mice fed normal chow (Fig. 5A, C respectively), with some vessels showing marked loss of tight junction protein expression (denoted by arrows in Fig. 5A, C), and quantified in Fig. 5B, D (p < 0.05). Analysis of the olfactory bulb, cerebral cortex and cerebellum revealed similar vascular loss of ZO-1 expression in PLX5622-fed mice exposed to CMH (Additional file 6: Figure S6; arrows denote loss of ZO-1 expression). These findings demonstrate that absence of microglia leads to increased BBB disruption, underscored by greater loss of endothelial tight junction proteins.

### CMH-induced cerebrovascular angiogenesis shows marked regional differences

**Fig. 6:**
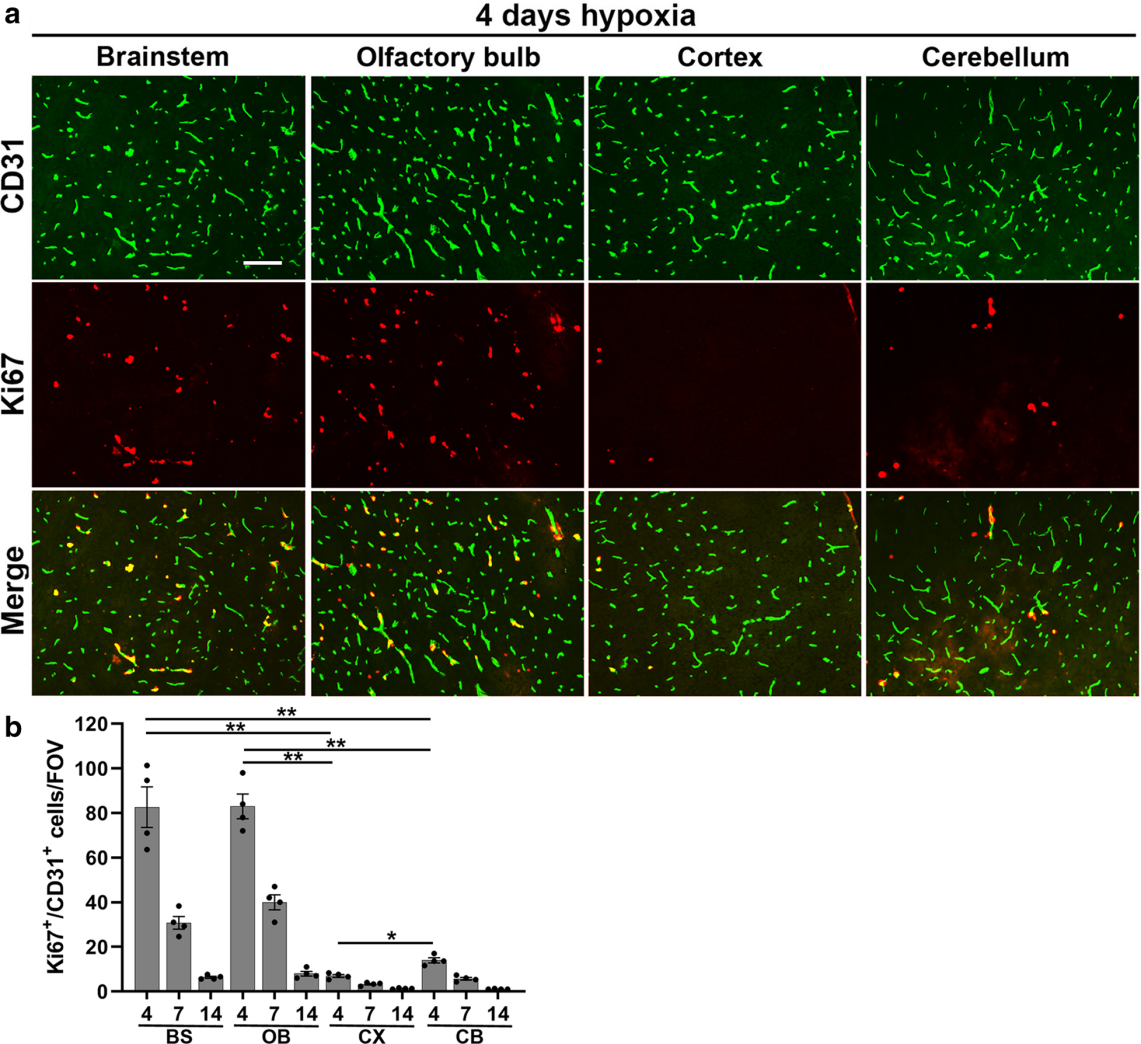
Levels of hypoxia-induced endothelial proliferation are region-specific. **a** Frozen brain sections taken from mice exposed to hypoxia (8% O_2_) for 4 days were stained for CD31 (AlexaFluor-488) and the proliferation marker Ki67 (Cy-3). Images were captured in the brainstem, olfactory bulb, cerebral cortex, and cerebellum. Scale bar = 100 μm. **b** Quantification of the number of proliferating endothelial cells (CD31 + /Ki67 + cells)/FOV. Results are expressed as the mean ± SEM (n = 4 mice/group). *p < 0.05; **p < 0.01. Note that hypoxia-induced endothelial proliferation was significantly higher in the brainstem and olfactory bulb versus the cerebral cortex and cerebellum

Based on our previous observation that hypoxia-induced vascular remodelling occurs in a similar time frame as the vascular leak we describe here [17, 27], we wondered if the vascular remodelling process which involves neighbouring endothelial cells temporarily uncoupling from each other, might be directly triggering these transient vascular leaks. To examine this relationship, we quantified the levels of brain endothelial proliferation in the two brain regions showing high (brainstem and olfactory bulb) and low (cerebral cortex and cerebellum) levels of vascular leak. As shown in Fig. 6A (and quantified in Fig. 6B), all four regions demonstrated that the highest level of endothelial proliferation occurred after 4 days hypoxia, and gradually declined at later timepoints (days 7 and 14), consistent with previous findings [26, 27]. What was most striking was that CMH stimulated much higher levels of endothelial proliferation in the brainstem and olfactory bulb compared to the cerebral cortex and cerebellum (*p* < 0.01). This data demonstrates a strong correlation between CMH-induced endothelial proliferation and vascular disruption, supporting the concept that vascular remodelling either directly or indirectly, leads to increased risk of transient vascular leak.

## Discussion

In a recent study we highlighted a key role for microglia in protecting and repairing hypoxia-induced vascular leaks in spinal cord blood vessels [17]. As blood vessels in the spinal cord and brain share many similarities, the goal of the current study was to determine if microglia play a similar vasculo-protective role in the hypoxic brain. Our main findings were as follows: (1) as in the spinal cord, chronic mild hypoxia (CMH) triggered transient vascular leak in cerebral blood vessels, which was strongly associated with aggregation and activation of microglia around disrupted vessels, (2) CMH-induced vascular leak showed regional selectivity, being much more prevalent in the brainstem and olfactory bulb than the cerebral cortex and cerebellum, (3) depletion of microglia with the CSF-1R inhibitor PLX5622, had no effect under normoxic conditions, but markedly increased hypoxia-induced cerebrovascular leak in all brain regions examined, (4) in all brain regions, vascular leak was associated with increased expression of MECA-32, a marker of compromised CNS endothelium, and greater endothelial loss of tight junction proteins, (5) this correlated with astrocyte-vascular uncoupling, as defined by vascular loss of AQP4 in brain regions showing highest vascular leak, but not in regions showing less leak, and (6) CMH-induced angiogenic remodeling was much higher in regions showing high vascular leak (brainstem and olfactory bulb), demonstrating a strong association between the extent of hypoxia-induced angiogenic remodeling and vascular leak. Taken together, our findings confirm that CMH triggers transient vascular leak in the brain just as it does in the spinal cord, which then provokes a microglial vasculo-protective response. Our work also suggests that transient BBB disruption may be an unwanted side-effect of hypoxic-induced angiogenic remodeling.

In a similar manner to the spinal cord, CMH triggered vascular disruption in only a small fraction of blood vessels in the brain [17]. As the time sequence of hypoxic-induced vascular leak correlates closely with the timing of angiogenic remodeling [17, 27], it seems plausible that the process of endothelial proliferation and separation from neighboring cells, triggers a transient break in the BBB, which then results in vascular leak. Interestingly, we observed that the degree of susceptibility to vascular leak is very region-dependent, with greater numbers of leaks occurring in the olfactory bulb and brainstem, but far fewer in the cerebral cortex and cerebellum. What accounts for these striking differences is currently unclear. While it could be a result of intrinsic regional differences in endothelial cell properties, a number of other factors could equally contribute to this phenomenon, including local metabolic oxygen demands, cell (particularly neuronal) densities, anatomical and physical properties of blood vessels, and region-specific differences in supporting cells such as astrocytes, microglia and pericytes. Interestingly, our analysis of angiogenic remodeling, as measured by the number of proliferating endothelial cells in different regions, demonstrated a very close correlation between the extent of vascular remodeling and vascular leak, strongly suggesting that transient BBB disruption may be an unwanted side-effect of hypoxic-induced angiogenic remodeling. In future studies it will be interesting to inhibit hypoxia-induced angiogenic remodeling and evaluate whether this prevents hypoxia-induced vascular leak. In principle, this could be achieved using a genetic approach by blocking cell-specific hypoxia-inducible factor-1α (HIF-1α) and/or HIF-2α signaling using Cre-Lox based transgenic mice [7, 39], though as the cellular origin of these signaling pathways in driving cerebrovascular remodeling has yet to be fully defined, it will first be essential to characterize these pathways. Our findings also demonstrated that brain regions showing high vascular leak correlated with more astrocyte-vascular uncoupling (defined by loss of AQP4 expression), raising the possibility that the ability to maintain astrocyte-vascular coupling is an important predictor of vascular integrity.

Our findings add to the growing body of evidence that microglia make an important contribution to the regulation of BBB integrity. It is well established that the fundamental cellular and molecular components of the BBB include inter-endothelial tight junction proteins (e.g.; ZO-1, occludin and claudin-5), adherens proteins (e.g.; vascular endothelial (VE)-cadherin and the junctional adhesion molecules (JAMs)), the vascular basement membrane extracellular matrix (ECM) proteins laminin and collagen IV, as well as the influence of astrocyte endfeet and pericytes [1, 9, 11, 18, 19, 33]. This study confirmed previous findings that CMH results in enhanced cerebrovascular expression of tight junction proteins [16, 27], though the signaling mechanisms underlying this change have yet to be defined. Interestingly, this upregulation was flattened in microglia-depleted mice, suggesting that either the enhanced degree of vascular leak results in degradation of tight junction proteins, or that microglia signaling events are required to mediate this response. In future studies, we aim to define which of these mechanisms is responsible. In terms of microglial contributions, while a pathogenic role for chronically activated pro-inflammatory microglia has been implicated in BBB disruption during ischemic stroke and intracerebral hemorrhage [21, 29], perhaps in part mediated by ECM-degrading matrix metalloproteinases (MMPs) [4, 42], only very recently have vasculo-protective functions of microglia been described, specifically in laser ablation, ischemic stroke and diabetic mouse models [20, 30, 38]. Our hypoxic studies, both recently in the spinal cord [17] and those described here in the brain, confirm that microglia play an important vasculo-protective function in maintaining CNS vascular stability. One caveat to consider is that as PLX5622 also depletes perivascular macrophages, it is also possible that this cell type may also contribute to vascular protection from hypoxic insult. In ongoing studies, we are currently investigating this possibility.

Declining levels of BBB integrity have been well described both during the course of normal aging, and in vascular dementia, more properly described as vascular contributions to cognitive impairment and dementia (VCID) [5, 14, 36]. This raises the important questions: why does BBB integrity decline with age and what mechanisms counteract this deterioration? Our current studies offer two clues that may be relevant to answering these questions: first, chronic exposure to hypoxia results in BBB disruption, implying that repeated hypoxic insults could lead to cumulative BBB damage in the aged, and thereby pre-dispose to VCID, and second, microglia play a critical vasculo-protective role in counteracting hypoxia-induced leak. As hypoxia is experienced in a wide variety of different medical conditions, including obstructive sleep apnea, lung disease (chronic obstructive pulmonary disease (COPD) and asthma), and age-related cardiac and cerebrovascular insufficiency, many of which are strong risk factors for cognitive decline [6, 25, 35, 40, 41], our findings have obvious implications for the harmful role of hypoxic insult in the pathogenesis of cerebrovascular leak and its contribution to VCID. Based on these findings, our working hypothesis is that in the young, any hypoxic insults (asthma or high altitude) have only minimal impact because the vasculo-protective mechanisms (including microglia) are efficient and quickly repair vascular defects. However in the aged brain, as hypoxic insults start to become more numerous and more severe (due to age-related deterioration of cardiorespiratory systems), vascular defects start to accumulate, resulting in loss of BBB integrity, disruption of cerebral homeostasis, neuronal death, and the appearance of cerebral micro-infarcts and cognitive decline. As BBB integrity gradually declines with age [5, 14, 36], the goal of future studies will be to understand whether this is due to intrinsic deterioration of vascular structure/function per se or due to declining microglial vasculo-protective activity. By gaining a more complete understanding of the molecular mechanisms regulating microglial vasculo-protection, and the impact of aging on this process, we will be better placed to manipulate microglial behavior in the aged brain to optimize this important function. Such an approach could have strong therapeutic potential in the prevention and treatment of VCID.

## Supplementary information

Additional file 1: Figure S1. Mac-1+ cell surrounding the leaking vessels also express Tmem119. Frozen brain sections taken from mice maintained under hypoxic conditions for 7 days were stained for Mac-1 (Cy-3) and Tmem119 (AlexaFluor-488). Scale bar = 50 μm. Note the strong co-localization of Mac-1 and Tmem119.

Additional file 2: Figure S2. Microglial depletion results in greater vascular leak in all brain regions examined during CMH. Frozen brain sections taken from mice fed normal chow or PLX5622-containing chow and maintained under hypoxic conditions for 7 days were stained for CD31 (AlexaFluor-488) and fibrinogen (Cy-3). Scale bar = 200 μm. Note that in all brain regions examined (olfactory bulb, cerebral cortex and cerebellum), PLX5622-treated mice showed a much higher number of leaky blood vessels compared with normal chow-fed controls.

Additional file 3: Figure S3. Quantification of the number of microglia surrounding leaking blood vessels. A. Frozen brain sections taken from mice maintained under hypoxic conditions for 7 days were stained for Mac-1 (AlexaFluor-488), fibrinogen (Cy-3) and the nuclear stain DAPI (blue). Scale bar = 50 μm. B. Quantification of the number of microglia congregating at the leakage site. Results are expressed as the mean ± SEM (n = 4 mice). Note that the number of microglia congregating at the leakage site ranged from 1 to 9, with an average of 4 microglia per vascular leak.

Additional file 4: Figure S4. Under hypoxic conditions absence of microglia results in region-specific astrocyte-vascular uncoupling. Frozen brain sections taken from mice fed normal chow or PLX5622-containing chow and maintained under hypoxic conditions for 7 days were stained for CD31 (AlexaFluor-488) and AQP4 (Cy-3). Scale bar = 50 μm. Note that under hypoxic conditions, PLX5622-fed mice showed a significant number of cerebral blood vessels in the olfactory bulb that lacked AQP4 expression (see arrows), though AQP4 loss was not observed in the cerebral cortex or cerebellum.

Additional file 5: Figure S5. Under hypoxic conditions, absence of microglia results in increased vascular MECA-32 expression in all brain regions examined. Frozen brain sections taken from mice fed normal chow or PLX5622-containing chow and maintained under hypoxic conditions for 7 days were stained for CD31 (AlexaFluor-488) and MECA-32 (Cy-3). Scale bar = 50 μm. Note that in mice fed PLX5622, all brain regions examined (olfactory bulb, cerebral cortex and cerebellum) showed a higher number of vessels expressing MECA-32.

Additional file 6: Figure S6. Microglial depletion results in greater loss of endothelial tight junction protein expression during CMH in all brain regions examined. Frozen brain sections taken from mice fed normal chow or PLX5622-containing chow and maintained under hypoxic conditions for 7 days were stained for CD31 (AlexaFluor-488) and ZO-1 (Cy-3). Scale bar = 50 μm. Note that under hypoxic conditions, all brain regions examined (olfactory bulb, cerebral cortex and cerebellum) in PLX5622-fed showed focal areas in which blood vessels showed diminished expression of ZO-1 (see arrows).

## Data Availability

The datasets used and/or analysed during the current study are available from the corresponding author on reasonable request.

## Abbreviations

AQP4: aquaporin 4
BBB: blood–brain barrier
BS: brain stem
CB: cerebellum
CMH: chronic mild hypoxia
COPD: chronic obstructive pulmonary disease
CNS: central nervous system
CSF-1R: colony stimulating factor-1 receptor
CX: cerebral cortex
Dual-IF: dual-immunofluorescence
EC: endothelial cell
ECM: extracellular matrix
FOV: field of view
JAMs: junctional adhesion molecules
MECA-32: mouse endothelial cell antigen-32
MMP: matrix metalloproteinase
MS: multiple sclerosis
OB: olfactory bulb
SEM: standard error of the mean
VCID: vascular contributions to cognitive impairment and dementia
ZO-1: zonula occludens-1
