# Supplementary figures and images for: Mild hypoxia triggers transient blood–brain barrier disruption: a fundamental protective role for microglia
Source: Acta Neuropathol Commun. 2020 Oct 28;8:175. doi: 10.1186/s40478-020-01051-z (PMC7592567; doi:10.1186/s40478-020-01051-z)

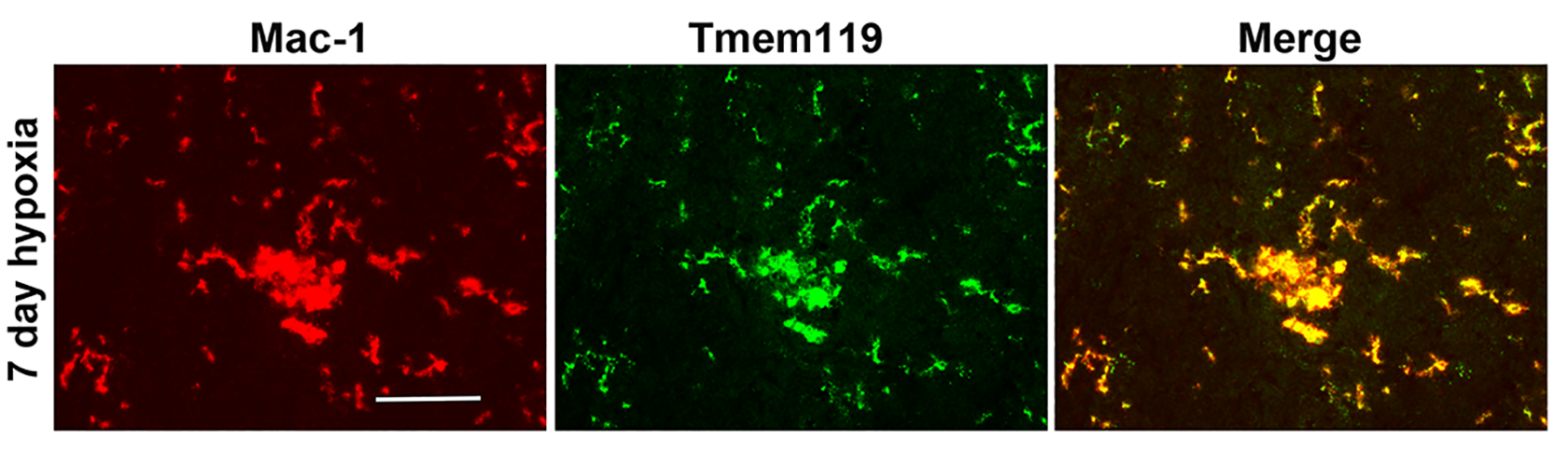

Supplement: Supplementary file 1 — Mac-1+ cell surrounding the leaking vessels also express Tmem119. Frozen brain sections taken from mice maintained under hypoxic conditions for 7 days were stained for Mac-1 (Cy-3) and Tmem119 (AlexaFluor-488). Scale bar = 50 μm. Note the strong co-localization of Mac-1 and Tmem119. [file 40478_2020_1051_MOESM1_ESM.tif]

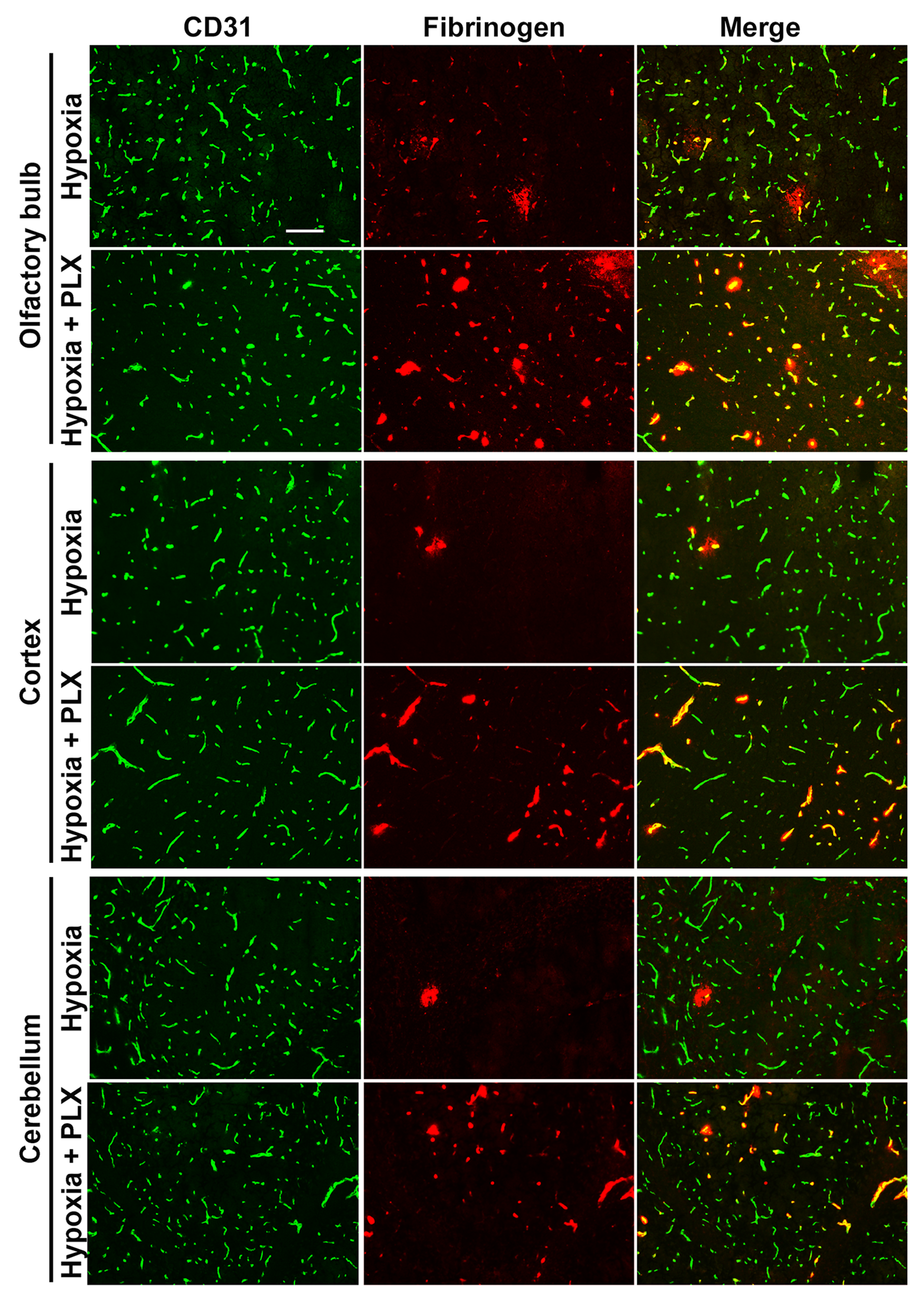

Supplement: Supplementary file 2 — Microglial depletion results in greater vascular leak in all brain regions examined during CMH. Frozen brain sections taken from mice fed normal chow or PLX5622-containing chow and maintained under hypoxic conditions for 7 days were stained for CD31 (AlexaFluor-488) and fibrinogen (Cy-3). Scale bar = 200 μm. Note that in all brain regions examined (olfactory bulb, cerebral cortex and cerebellum), PLX5622-treated mice showed a much higher number of leaky blood vessels compared with normal chow-fed controls. [file 40478_2020_1051_MOESM2_ESM.tif]

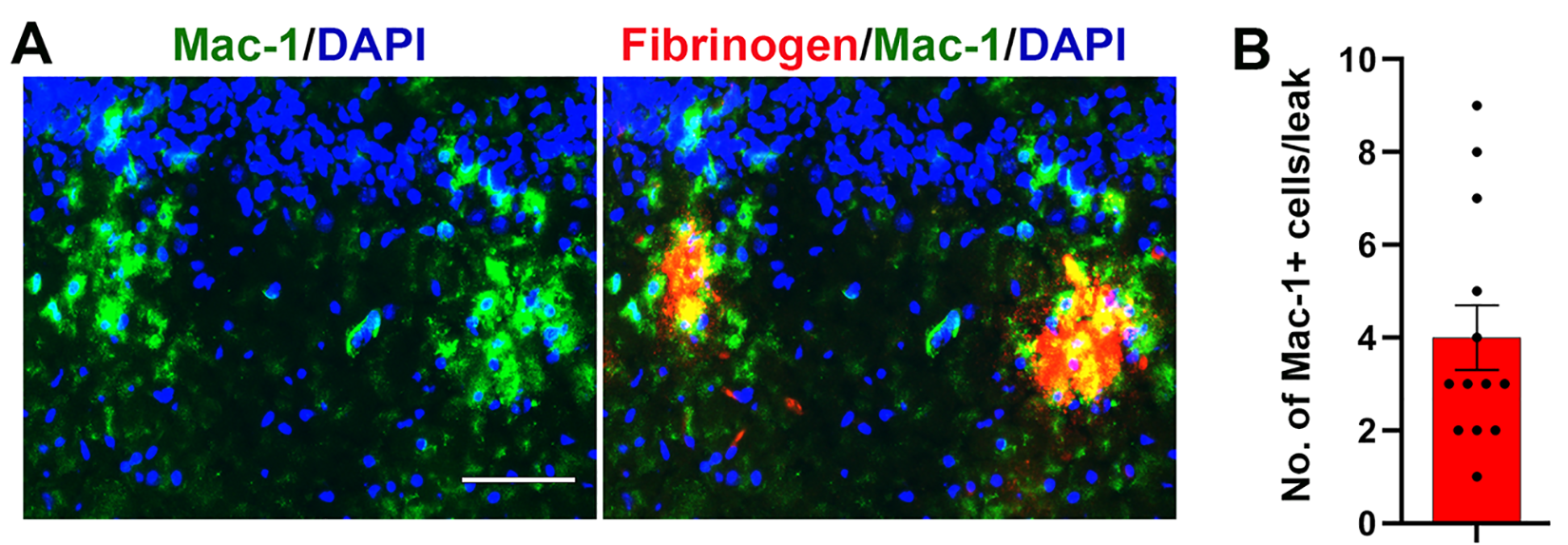

Supplement: Supplementary file 3 — Quantification of the number of microglia surrounding leaking blood vessels. A. Frozen brain sections taken from mice maintained under hypoxic conditions for 7 days were stained for Mac-1 (AlexaFluor-488), fibrinogen (Cy-3) and the nuclear stain DAPI (blue). Scale bar = 50 μm. B. Quantification of the number of microglia congregating at the leakage site. Results are expressed as the mean ± SEM (n = 4 mice). Note that the number of microglia congregating at the leakage site ranged from 1 to 9, with an average of 4 microglia per vascular leak. [file 40478_2020_1051_MOESM3_ESM.tif]

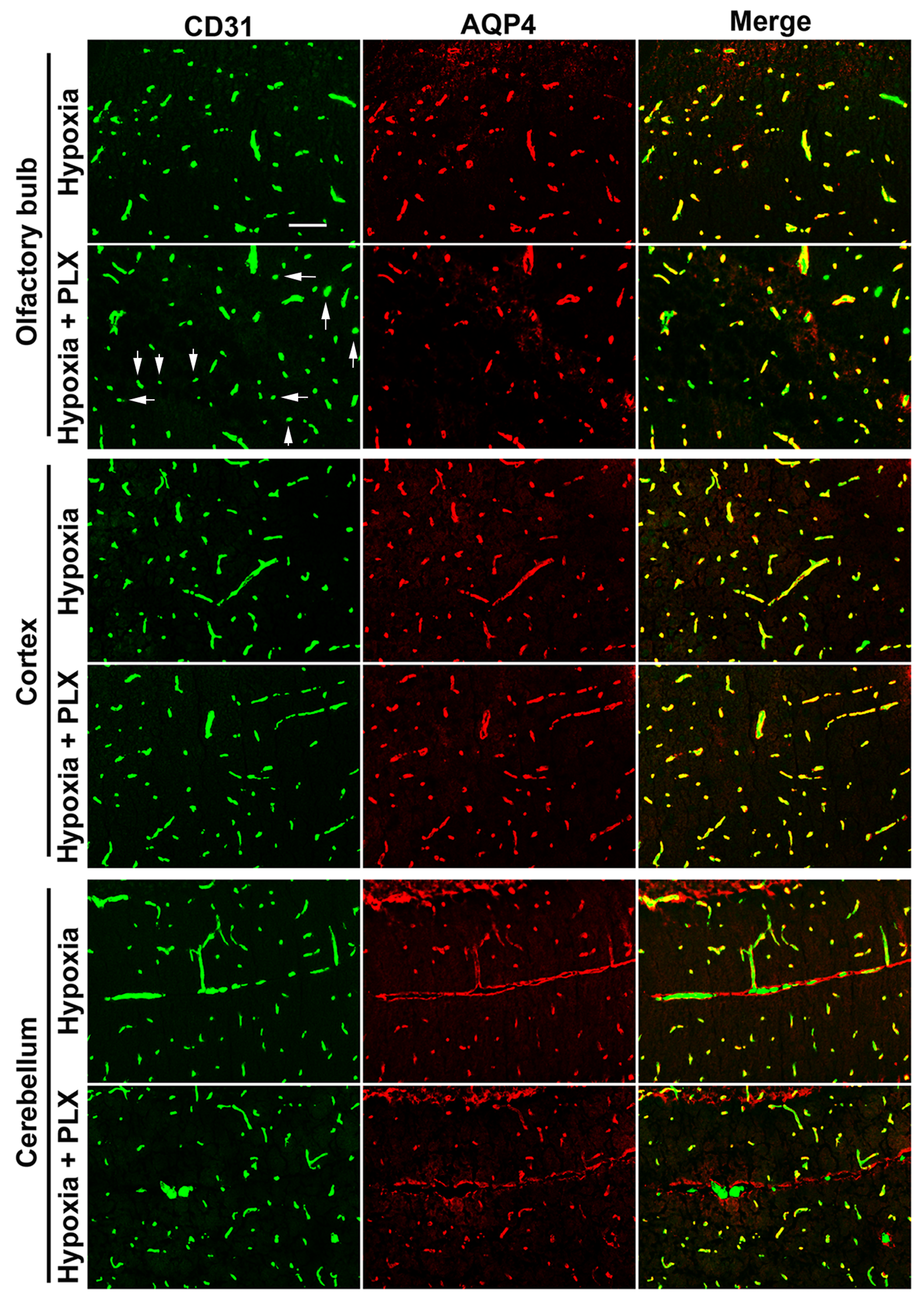

Supplement: Supplementary file 4 — Under hypoxic conditions absence of microglia results in region-specific astrocyte-vascular uncoupling. Frozen brain sections taken from mice fed normal chow or PLX5622-containing chow and maintained under hypoxic conditions for 7 days were stained for CD31 (AlexaFluor-488) and AQP4 (Cy-3). Scale bar = 50 μm. Note that under hypoxic conditions, PLX5622-fed mice showed a significant number of cerebral blood vessels in the olfactory bulb that lacked AQP4 expression (see arrows), though AQP4 loss was not observed in the cerebral cortex or cerebellum. [file 40478_2020_1051_MOESM4_ESM.tif]

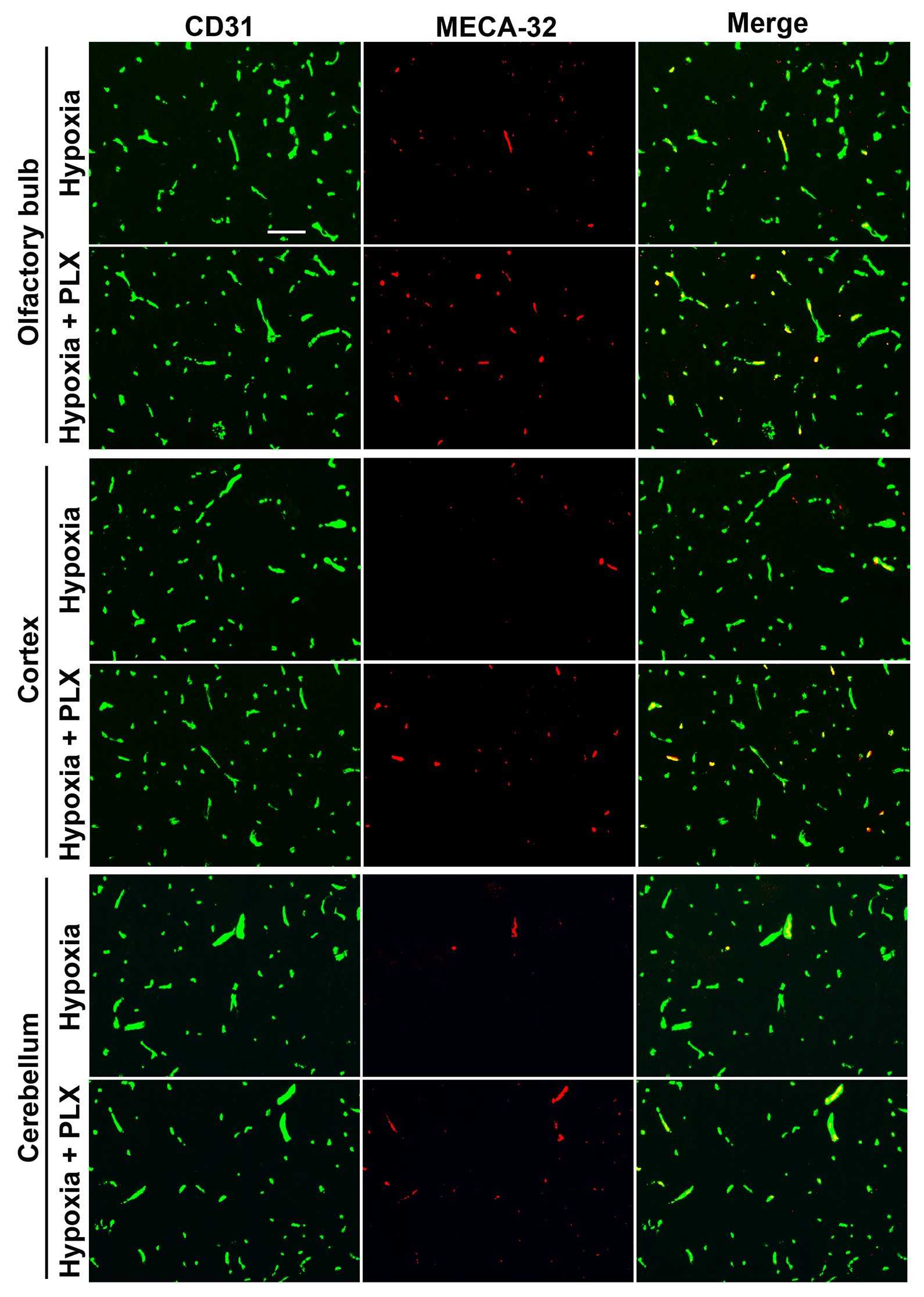

Supplement: Supplementary file 5 — Under hypoxic conditions, absence of microglia results in increased vascular MECA-32 expression in all brain regions examined. Frozen brain sections taken from mice fed normal chow or PLX5622-containing chow and maintained under hypoxic conditions for 7 days were stained for CD31 (AlexaFluor-488) and MECA-32 (Cy-3). Scale bar = 50 μm. Note that in mice fed PLX5622, all brain regions examined (olfactory bulb, cerebral cortex and cerebellum) showed a higher number of vessels expressing MECA-32. [file 40478_2020_1051_MOESM5_ESM.tif]

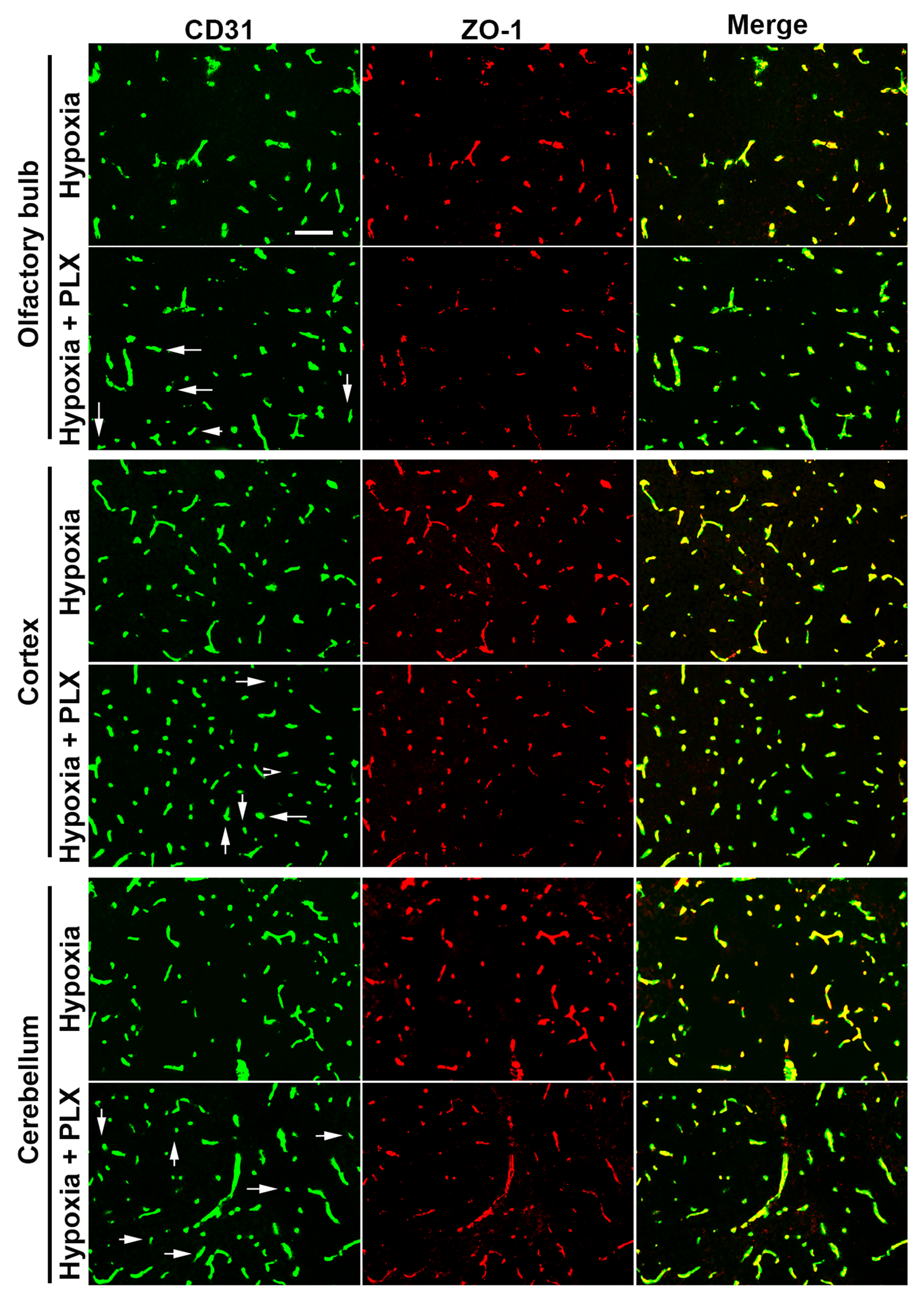

Supplement: Supplementary file 6 — Microglial depletion results in greater loss of endothelial tight junction protein expression during CMH in all brain regions examined. Frozen brain sections taken from mice fed normal chow or PLX5622-containing chow and maintained under hypoxic conditions for 7 days were stained for CD31 (AlexaFluor-488) and ZO-1 (Cy-3). Scale bar = 50 μm. Note that under hypoxic conditions, all brain regions examined (olfactory bulb, cerebral cortex and cerebellum) in PLX5622-fed showed focal areas in which blood vessels showed diminished expression of ZO-1 (see arrows). [file 40478_2020_1051_MOESM6_ESM.tif]
